# Rare association of acromegaly with left atrial myxoma in Carney's complex due to novel *PRKAR1A* mutation

**DOI:** 10.1530/EDM-14-0023

**Published:** 2014-09-01

**Authors:** Shweta Birla, Sameer Aggarwal, Arundhati Sharma, Nikhil Tandon

**Affiliations:** 1 Laboratory of Cyto-Molecular Genetics, Department of Anatomy, All India Institute of Medical Sciences, New Delhi, India; 2 Department of Endocrinology and Metabolism, All India Institute of Medical Sciences, New Delhi, India

## Abstract

Carney complex (CNC) is a rare autosomal dominant syndrome characterized by pigmented lesions of the skin and mucosae along with cardiac, endocrine, cutaneous, and neural myxomatous tumors. Mutations in the *PRKAR1A* gene have been identified in ∼70% of the CNC cases reported worldwide. A 30-year-old male was referred to the endocrinology clinic with suspected acromegaly. He had a history of recurrent atrial myxoma for the past 8 years for which he underwent repeated surgeries. Presently, he complained of having headache, excessive snoring, sweating, and also noticed increase in his shoe size. Evaluation for acromegaly revealed elevated levels of GH in random as well as in suppressed condition. Magnetic resonance imaging scan revealed enlarged sella with microadenoma in the left anterior pituitary. Screening of *PRKAR1A* gene was carried out for the patient, his parents and siblings who were available and willing to undergo the test. The patient was diagnosed to have the rare CNC syndrome characterized by recurrent atrial myxoma and acromegaly due to a novel 22 bp insertion mutation in *PRKAR1A* which was predicted to be deleterious by *in silico* analysis. Screening the available family members revealed the absence of this mutation in them except the elder brother who also tested positive for this mutation. The present study reports on a novel *PRKAR1A* insertion mutation in a patient with acromegaly and left atrial myxoma in CNC.

**Learning points:**

- Identification of a novel deleterious *PRKAR1A* insertion mutation causing CNC.
- It is important that patients with cardiac myxoma be investigated for presence of endocrine overactivity suggestive of CNC.
- *PRKAR1A* mutation analysis should be undertaken in such cases to confirm the diagnosis in the patients as well as first degree relatives.
- This case highlights an important aspect of diagnosis, clinical course, and management of this rare condition.

## Background

Carney complex (CNC) is a rare syndrome characterized by neoplasia involving the heart, CNS, and endocrine organs (1). It is a genetically heterogeneous syndrome and is inherited as an autosomal dominant trait. The regulatory subunit (R1A) of the protein kinase A (*PRKAR1A*) gene is implicated in its causation.

*PRKAR1A* (OMIM # 188830) is a tumor suppressor gene that encodes a protein kinase A (PKA) regulatory 1-alpha subunit and is located on chromosome 17q24. The *PRKAR1A* mutations causing CNC have been identified in ∼70% of the total cases reported worldwide (2). Majority of *PRKAR1A* mutations are reported to be nonsense, frameshift, and splicesite, which undergo nonsense-mediated mRNA decay (NMD) with resultant haploinsufficiency (3).

We report here a novel deleterious genetic finding in a patient with CNC who presented with recurrent left atrial myxoma and acromegaly.

## Case presentation

A 30-year-old male, surgically operated for recurrent left atrial myxoma, presented to the Endocrine out-patient department of the All India Institute of Medical Sciences, New Delhi, with suspected acromegaly.

The history of present illness goes back to 2004 when the patient developed an episode of left hemiparesis and left upper motor neurone facial nerve palsy. He was managed at another medical care facility and recovered within a week. Echocardiography revealed left atrial myxoma (45×20 mm) for which he was operated in same year, followed by a repeat surgery after 1 year for a recurrence. The patient was on irregular follow-up after the second surgery, till the year 2011 when he was referred to the endocrine clinic for suspected acromegaly.

There was no history suggestive of increase in acral size, headache, galactorrhea, abdominal striae, easy bruisaibility, proximal weakness, skin pigmentation, nor any family history of such illness.

### Clinical investigation

Cardiac examination revealed normal sinus rhythm with heart rate of 90/min, blood pressure of 150/70 mmHg, and a mid systolic click was audible in mitral area. Acanthosis nigricans was present in axilla and neck.

**Figure 1:**
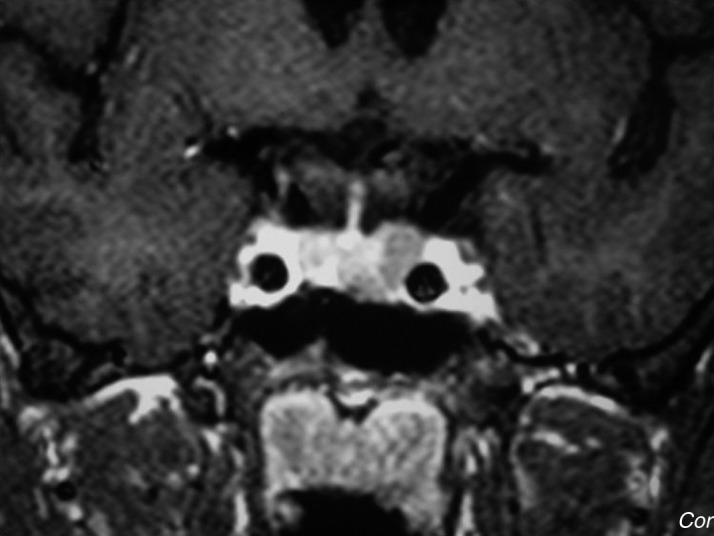
MRI sella showing left pituitary microadenoma.

Echocardiography showed left atrial myxoma (2.2×2.4 cm) attached to the inter atrial septum. Hormonal investigation showed prolactin level of 21.5 ng/ml (normal range 4.6–21.4 ng/ml) and total T_4_ level of 6.4 μg/dl (normal range 5.1–14.1 μg/dl). Dynamic testing of the pituitary axis revealed growth hormone values of 16 ng/ml at baseline, and 12.6 ng/ml, 60 min after administering 75 g glucose orally (normal growth hormone post 75 g glucose challenge should be <1 ng/ml). Morning serum cortisol level was 8 μg/dl (normal range 6.2–19.2 μg/dl) and salivary cortisol value at 2300 h was 0.1 μg/dl (normal <0.43 μg/dl). Magnetic resonance imaging (MRI) of sella (Fig. 1) showed pituitary microadenoma (9×6 mm) of the left half anterior pituitary. Contrast enhanced CT of the adrenal area revealed normal sized adrenal glands.

The patient underwent pituitary surgery through transnasal transsphenoidal route. Histopathology and immunohistochemistry showed growth hormone staining pituitary adenoma. Post-operative follow-up at 5 months after surgery showed growth hormone levels to be 0.65 ng/ml fasting basal and 0.58 ng/ml 1 h after 75 g glucose load. Pre and post-operative IGF1 values were 599 and 173 ng/ml respectively (normal reference range from 26 to 30 years 75 to 275 ng/ml).

### Genetic screening

**Figure 2:**
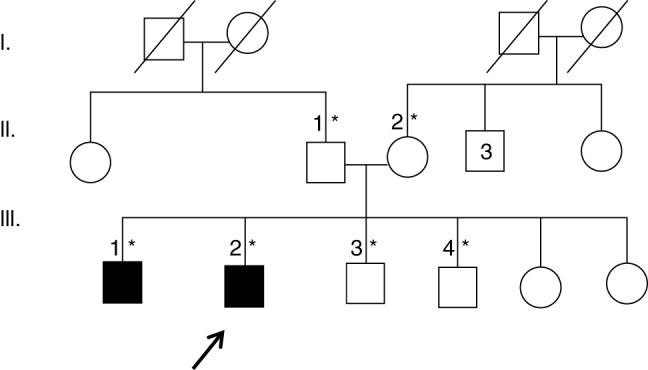
Pedigree of the affected individual with recurrent atrial myxoma/acromegaly/novel 22 bp insertion mutation in *PRKAR1A*. Roman numerals indicate the generation. Asterisk (*) indicates the individuals who were screened for the mutation of *PRKAR1A* gene. Squares represent males and females are denoted by circles. Arrow indicates the proband. Open boxes represent unaffected individuals. Arabic numerals are the individuals discussed in the text.

Detailed family history and pedigree information was collected (Fig. 2). Peripheral blood sample was drawn in EDTA tubes for molecular investigations after taking informed consent from the patient, his parents, and siblings who were available and willing to undergo the test. Genomic DNA was extracted using standard salting out protocol and subjected to PCR amplification of the *PRKAR1A* gene with primers described previously (2) using 70–100 ng DNA, 1.25 mM MgCl_2_, 0.25 mM of each of the dNTPs (Invitrogen), 5 pM of each primer and 0.5 units of Taq Polymerase (Invitrogen) in a 25 μl volume mixture using thermocycler ABI 9700 (Applied Biosystems).

### Sequencing and mutation analysis

All the amplified products were purified using the Qiagen kits (Qiagen, GmbH), sequenced using BigDye Terminator Mix version 3.1 (Applied Biosystems) and analyzed on an ABI-3100 Genetic Analyzer (Applied Biosystems). The nucleotide sequences were compared with the published cDNA sequences of *PRKAR1A* (GenBank accession number ENSG00000108946) gene.

### *In silico* analysis

Prediction of functional effects of the novel mutation was done using softwares PROVEAN (http://provean.jcvi.org/index.php) and MutationT@ster (http://www.mutationtaster.org/) (4) (5).

## Investigation

**Figure 3:**
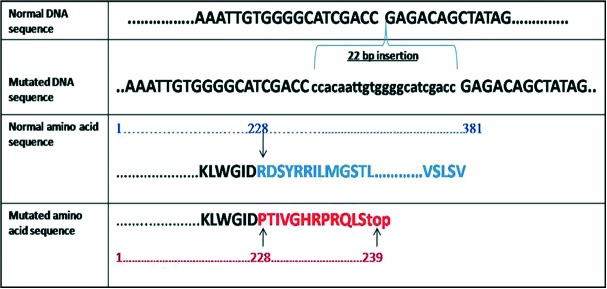
Heterozygous insertion of 22 bp in exon 6 disrupting codon 228, leading to frameshift downstream and creating STOP codon at 239 position.

Direct sequencing of the *PRKAR1A* gene in the patient revealed a heterozygous 22 bp insertion mutation in exon 6 (Fig. 3). Screening of the available family members revealed the absence of this mutation in them except the elder brother who is positive for this mutation.

Assessment for pathogenicity using PROVEAN showed it to be deleterious (score −19.027) and MutationT@ster predicted it to be disease causing (score=1) leading to frameshift thereby causing splice site changes which might have resulted in affecting the protein features and NMD.

## Discussion

CNC, as described by J Aiden Carney in 1985, is an autosomal dominant disorder characterized by neoplasia involving heart, CNS, and endocrine organs. Presence of pigmented skin and mucosal lesions along with these tumors is an important hallmark of this syndrome (1) (6). Most of the cases are familial and the median age of presentation is 20 years. CNC can manifest itself as spotty cutaneous pigmentation, cutaneous myxomas, cardiac myxomas, psammomatous melanotic schwanoma, acromegaly, large cell calcifying Sertoli cell tumor, thyroid carcinomas or nodules, and breast adenomas (7).

The most common endocrine gland manifestations are acromegaly, thyroid and testicular tumors, and adrenocorticotropic hormone-independent Cushing's syndrome due to primary pigmented nodular adrenocortical disease (PPNAD).

Cushing's syndrome due to PPNAD is observed in children and young adults, with a peak during the second decade of life. It is rare but can occur before the age of 4 years and it is rarely diagnosed after the age of 40 years. Diagnosis of Cushing's syndrome due to PPNAD is often difficult because hypercortisolism can develop progressively over years (3).

It is generally established that a patient presenting with two or more of the manifestations given in Table 1 would be diagnosed as having CNC. If the patient has a germline *PRKAR1A* mutation and/or a first-degree relative affected by CNC, a single manifestation is sufficient for the diagnosis (3).

**Table 1:** Showing features of Carney complex

**Manifestations of Carney complex in affected individuals**	**Percentage**
Primary pigmented nodular adrenal cortical disease (PPNAD)	25–60
Cardiac myxoma	30–60
Skin myxoma	20–63
Lentiginosis	60–70
Multiple blue nervus	
Breast ductal adenoma	25
Testicular tumours	33–56
Ovarian cyst	20–67
Acromegaly	10
Thyroid tumour	10–25
Melanotic schwannoma	8–18
Osteochondrotic myxoma	<10

*PRKAR1alpha* is a tumor suppressor gene, which is found to be mutated in almost 50% of CNC cases demonstrating an autosomal dominant transmission (8). When a *PRKAR1A* mutation is identified, genetic analysis should be undertaken in first-degree relatives. Routine echocardiography screening of the first degree relatives is appropriate for searching for familial myxomas (8). Interestingly, in the present report, at the time of sampling for molecular studies the elder brother (III.1) was apparently normal and did not show any symptoms of CNC but only had a history of infertility similar to the index case (III.2). Shortly afterwards he had repeated episodes of embolization with hemiparesis. Echocardiography revealed bilateral atrial myxoma, for which he underwent surgery.

The cAMP–signaling pathway is crucial for normal functioning of the endocrine cells. Any disruption in this pathway may result in tumorigenesis. PKA, the main mediator of cAMP signalling, is a tetramer consisting of two regulatory and two catalytic subunits.

In humans, four different regulatory (*PRKAR1A*, *PRKAR1B*, *PRKAR2A*, and *PRKAR2B*) and three catalytic subunits (*PRKACA*, *PRKACB*, and *PRKACG*) have been identified till date. The two molecules of cAMP bind to the regulatory PKA dimer under high cAMP concentration in the cellular environment. This produces a reversible conformational change that disrupts the PKA tetramer and results in dissociation of its catalytic subunits. These dissociated catalytic subunits regulate phosphorylation of different effector enzymes, ion channels, and transcription of specific genes that mediate metabolism, cell proliferation, differentiation, and apoptosis (9). Maximum numbers of mutations have been found in *PRKAR1A*, one of the four regulatory PKA subunits (10). Majority of *PRKAR1A* mutations result in frameshift changes generating a premature stop codon. Such mutant mRNA molecules are unstable and are degraded by the process of NMD.

The insertion of heterozygous 22 bp exon 6 *PRKAR1A* identified in the patient disrupts codon 228, leading to frameshift downstream and creating a premature stop codon at position 239 in the mRNA which undergoes NMD. This results in 50% reduction in normal cellular PRKAR1A levels because one copy of the *PRKAR1A* gene is mutated and the other is normal. This reduction in regulatory component level results in a higher proportion of catalytic subunits causing increased signaling and activation of the downstream cellular processes of proliferation and differentiation leading to tumorigenesis.

The mutation identified in the index case was also seen in his elder brother (III.1). Absence of the mutation in the parental DNA suggests its *de novo* occurrence. Previous studies have reported high-frequency occurrence of sporadic cases with *de novo* *PRKAR1A* mutations (11). Several reports have described this as a heterogeneous disorder with the presence of small indels and large gene rearrangements (12).

The 22 bp insertion mutation found in this study is a duplication of an 18 bp region intervened by a stretch of four nucleotides. To the best of our knowledge, the sequence change is novel and is not reported or found associated with any other disorder.

To rule out the possibility of the change to be a polymorphism, 100 chromosomes from healthy individuals who acted as controls were screened for the presence of the same. The change was not identified in any of them, proving this to be a mutation and not a polymorphism. This is in concordance with the results of the *in silico* analysis which predicted the sequence change to be pathogenic resulting in the disease phenotype.

Infertility is an important phenotypic presentation in CNC cases. Previous studies have suggested the presence of several unknown reasons affecting fertility in CNC males other than Sertoli cell tumors (13). The report presents evidence for this important observation as infertility was present in both the brothers who were positive for the mutation. Clinical investigations ruled out the presence of Sertoli cells tumors in both.

Although the same mutation was identified in the two brothers, the elder one showed late onset of the disease as well as absence of acromegaly proving the heterogeneous nature of this condition. This is in accordance with other dominant disorders showing both inter- and intra-familial variable expressivity of the mutant allele.

## Conclusion

In conclusion, we have identified a novel deleterious *PRKAR1A* 22 bp insertion mutation in a familial case of CNC. The significance of this study is the identification of the new insertion mutation leading to CNC with unique clinical manifestations of infertility without Sertoli cell tumors, acromagaly, and cardiac myxoma. This novel change adds to the universal mutation pool of *PRKAR1A* causing CNC. The characteristic presentation documented in this family of infertility without Sertoli cell tumors along with acromagaly and cardiac myxoma in future will aid the clinicians in timely diagnosis and appropriate treatment management of this rare condition.

## Patient consent

Written informed consent has been obtained from the patient on the journal's consent form.

## Author contribution statement

S Birla carried out the molecular genetic studies, literature search, data analysis, and manuscript preparation. S Aggarwal was involved in the clinical diagnosis, literature survey, and manuscript preparation. A Sharma supervised the genetic studies and manuscript editing. Clinical diagnosis and management was performed under N Tandon's supervision.
